# A 30-year dataset of CO_2_ in flowing freshwaters in the United States

**DOI:** 10.1038/s41597-022-01915-0

**Published:** 2023-01-11

**Authors:** Timothy R. Toavs, Caleb T. Hasler, Cory D. Suski, Stephen R. Midway

**Affiliations:** 1 Department of Oceanography and Coastal Sciences, Louisiana State University, Baton Rouge, LA 70803 USA; 2 Department of Biology, The University of Winnipeg, Winnipeg, Manitoba Canada; 3 Department of Natural Resources, University of Illinois, Urbana, IL 61801 USA

**Keywords:** Projection and prediction, Environmental monitoring

## Abstract

Increasing atmospheric carbon dioxide (CO_2_) concentrations have been linked to effects in a wide range of ecosystems and organisms, with negative effects of elevated CO_2_ documented for marine organisms. Less is known about the dynamics of CO_2_ in freshwaters, but the potential exists for freshwater organisms to be challenged by elevated CO_2_. In flowing freshwaters CO_2_ exhibits more variability than in lakes or the ocean, yet spatiotemporally extensive direct measures of CO_2_ in freshwater are rare. However, CO_2_ can be estimated from pH, temperature, and alkalinity—commonly collected water quality metrics. We used data from the National Water Quality Monitoring Council along with the program PHREEQC to estimate CO_2_ in flowing freshwaters across 35,000 sites spanning the lower 48 US states from 1990 through 2020. Site data for water chemistry measurements were spatially joined with the National Hydrology Dataset. Our resulting dataset, CDFLOW, presents an opportunity for researchers to add CO_2_ to their datasets for further investigation.

## Background & Summary

Climate change caused by anthropogenically produced carbon dioxide (CO_2_) is an issue that poses challenges around the world, including within marine and freshwater ecosystems. CO_2_ concentrations in the atmosphere have been steadily increasing since the mid-nineteenth century with a total increase of around 40%^1^ in that time. While CO_2_ concentrations in the atmosphere have fluctuated throughout time, the rate of increase recorded since the 1850s is greater than any rate of increase that has occurred in the last million years^2^. As CO_2_ in the atmosphere rises, dissolution of CO_2_ into the ocean increases, thus interacting with the ocean carbonate system and ultimately leading to a decrease in ocean pH and a decrease in surface calcium carbonate (CaCO_3_) concentrations, a process known as ocean acidification^3^. Dissolved CO_2_ in marine and freshwater environments is measured as the partial pressure of CO_2_ (*p*CO_2_)^4^. This rise in *p*CO_2_ has been shown to affect a wide range of ecosystems and organisms, with negative effects of elevated *p*CO_2_ documented for marine and freshwater organisms. More specifically, ocean acidification caused by increasing atmospheric CO_2_ has been shown to alter fish behaviour and physiology^5^ and affect planktonic primary producers^6,7^. Outcomes of the effects are difficult to predict due to the variability across taxa. However, possible outcomes include reduced fish populations^5,8^ and declines in ocean primary productivity^9^. While the effects of elevated *p*CO_2_ in marine environments are well documented, less is known about the dynamics of *p*CO_2_ in freshwaters, but the potential exists for freshwater organisms to be challenged^10^.

While less is known about *p*CO_2_ dynamics in freshwater, some general characteristics and processes have been documented. Flowing freshwaters have many different potential sources of *p*CO_2_ and show high variability from one water body to another. Cole *et al*.^11^ showed that *p*CO_2_ in North American lakes was rarely at equilibrium with CO_2_ in the atmosphere and found a range of concentration differences from 175 times lower *p*CO_2_ than atmospheric CO_2_ to 57 times greater. Flowing freshwaters show more variability and are typically supersaturated compared to the atmosphere, and have even been identified as sources of atmospheric CO_2_^12^_._ Butman and Raymond^13^ verified supersaturation in US flowing freshwaters and found that there is a relationship between *p*CO_2_ and stream order suggesting a proportional relationship between stream size and *p*CO_2_. Typically, *p*CO_2_ in flowing freshwater is influenced by the water source of the flowing freshwater systems coupled with characteristics of that system including surrounding geologic conditions, *p*CO_2_ residence time, and the gas transfer velocity^14^. Other contributing factors to *p*CO_2_ include (but are not limited to) the balance between photosynthetic and respiration rates^15^ and terrestrial respiration^12^. No matter the source, flowing freshwaters display high variability in *p*CO_2_ and while not much is known about the potential impacts on freshwater organisms and ecosystems it is important to understand *p*CO_2_ spatiotemporal trends to identify potential impacts.

Considering the high variability displayed in flowing freshwaters a large spatiotemporal dataset is needed for understanding patterns and trends. Direct measures of *p*CO_2_ in flowing freshwaters are extremely limited making it challenging to define spatial or temporal *p*CO_2_ trends. However, *p*CO_2_ can be estimated from a combination of water quality metrics including pH, temperature, and alkalinity—commonly collected water quality metrics, and has been done numerous times throughout the literature^13,16–18^. Our dataset (referred to as CDFLOW)^19^ fills the need for a large spatiotemporal dataset using pH, temperature, and alkalinity measurements from across the lower 48 United States (CONUS) from 1990 through 2020. To our knowledge, CDFLOW^19^ is the largest publicly available *p*CO_2_ database with over 750,000 *p*CO_2_ estimates coming from over 35,000 sites. CDFLOW^19^ is also integrated with the National Hydrologic Dataset (NHD)^20^ allowing for the addition of other environmental and geospatial variables^21^ and ease when incorporating with other databases related to the NHD. CDFLOW^19^ provides an opportunity for spatiotemporal analysis of *p*CO_2_ across the CONUS and the possibility of adding *p*CO_2_ data to other researchers’ data.

## Methods

### Data query

Water quality measurements and their respective site-data (see below for site definition) were queried separately by each of the 48 CONUS states from the Water Quality Data Portal^22^ using the following filters:

- Country = “United States of America”
- Site Type = “Stream”
- Date Range from = “01-01-1990”
- Date Range to = “12-31-2020”
- Sample media = “Water”
- Characteristics = “Alkalinity, total”, “Alkalinity”, “pH”, and “Temperature, water”

The “Total alkalinity” and “Alkalinity” characteristic parameters are equivalent measurements but represent the different labels that respective reporting agencies use. The separate data queries for each state were merged using a shared variable called “MonitoringLocationIdentifier”. The data queries and subsequent data merges resulted in 48 water quality measurement datasets with matching site data, representing each state within the CONUS.

### *p*CO2 estimation

The 48 datasets were processed and formatted separately then combined into one dataset for estimating *p*CO_2_. The first step was to subset the datasets for quality and consistency among measurements. The following filters were applied:

- Removing non-numeric measurement values; e.g., “alkalinity <1 mg/l”
- Removing measurement values represented as statistical summaries and not observations; e.g., “average temp = 21 °C”
- Removing measurements not taken at the surface of the respective waterbody.
- Removing extreme water temperature measurements e.g., temperature ≤0 °C and temperature ≥40 °C
- Removing impossible pH values e.g., pH >14
- Removing pH values below 5.4

Hunt *et al*^23^. found that when pH is under 5.4 there is an increased risk of overestimating *p*CO_2_ due to the possibility of non-carbonate anions contributing to the total pH, thus filtering out pH values less than 5.4. pH over 14 was excluded because the standard pH scale goes from 0–14. No filters were applied to alkalinity measurements.

Next, we grouped temperature, pH, and alkalinity measurements by location, date, and time. Grouping was done by creating a key identification by concatenating the following columns: “MonitoringLocationIdentifier”, “ActivityStartDate”, and “ActivityStartTime”. If time data were not available for water quality measurements, they were still included but were grouped with water quality measurements also without time data. In grouping water quality measurements this way, they are grouped by the highest time/date resolution available, with day being the coarsest acceptable resolution. CDFLOW^19^ requires all three of the queried water quality metrics to be present in each group to estimate *p*CO_2_.

Finally, if a group had records of temperature, pH, and alkalinity, a single *p*CO_2_ value was estimated using the United States Geological Survey’s program PHREEQC v3^24^. PHREEQC quantitatively accounts for the chemical composition of a solution by relying on mole-balancing equations and in solving the mole-balance equations it derives the most likely *p*CO_2_ estimation^25^. It should be noted that PHREEQC calculates *p*CO_2_ under the assumption that alkalinity and pH in a system are determined by the current state of the carbonate system. PHREEQC can detect when this carbonate system assumption cannot be safely made in which case that group of observations was discarded. In cases where multiple measurements of a single water quality measurement were grouped with one or more of the two other required measurements, a measurement was chosen at random to be grouped for a *p*CO_2_ estimate. All measurements not grouped were then discarded. Also, we excluded extreme outliers in the *p*CO_2_ estimates which exceeded 2 standard deviations from the mean. The combination of the 48 processed, formatted, and estimated datasets resulted in a single dataset representing all our *p*CO_2_ estimates across the CONUS.

### Defining sites

The site data that was merged with water quality measurements included latitude and longitude coordinates. These coordinates corresponded with the location identifier for each water quality measurement, now a *p*CO_2_ estimate, and labeled as “MonitoringLocationIdentifier” (referred to as MID). We created a separate dataset using our dataset of *p*CO_2_ estimates across the CONUS created above, and this new dataset included each of the unique MIDs along with latitude and longitude coordinates. Using the dataset of unique MIDs, we spatially joined each unique MID with the Environmental Protection Agencies National Hydrological Dataset Plus V2^20,26^ (NHD) based on the closest stream catchment feature within NHD. Stream catchment features were labeled with a unique code called a COMID^27^. The spatial join resulted in a dataset with each unique MID now being associated with a COMID and was merged with our dataset of *p*CO_2_ estimates across the CONUS. We also calculated the distance between MID’s and the associated COMID, when the distance was greater than 100 meters the associated *pC*O_2_ estimate(s) was excluded from our dataset of *p*CO_2_ estimates across the CONUS. Finally, we spatially joined MID coordinates with Hydrologic Unit (HUC12)^28^ polygons included in the NHD. The result of the two spatial joins is the ability to group *p*CO_2_ estimates at any Hydrologic Units Code level and now sites within CDFLOW^19^ are defined as what COMID the estimate resides.

**Fig. 1:**
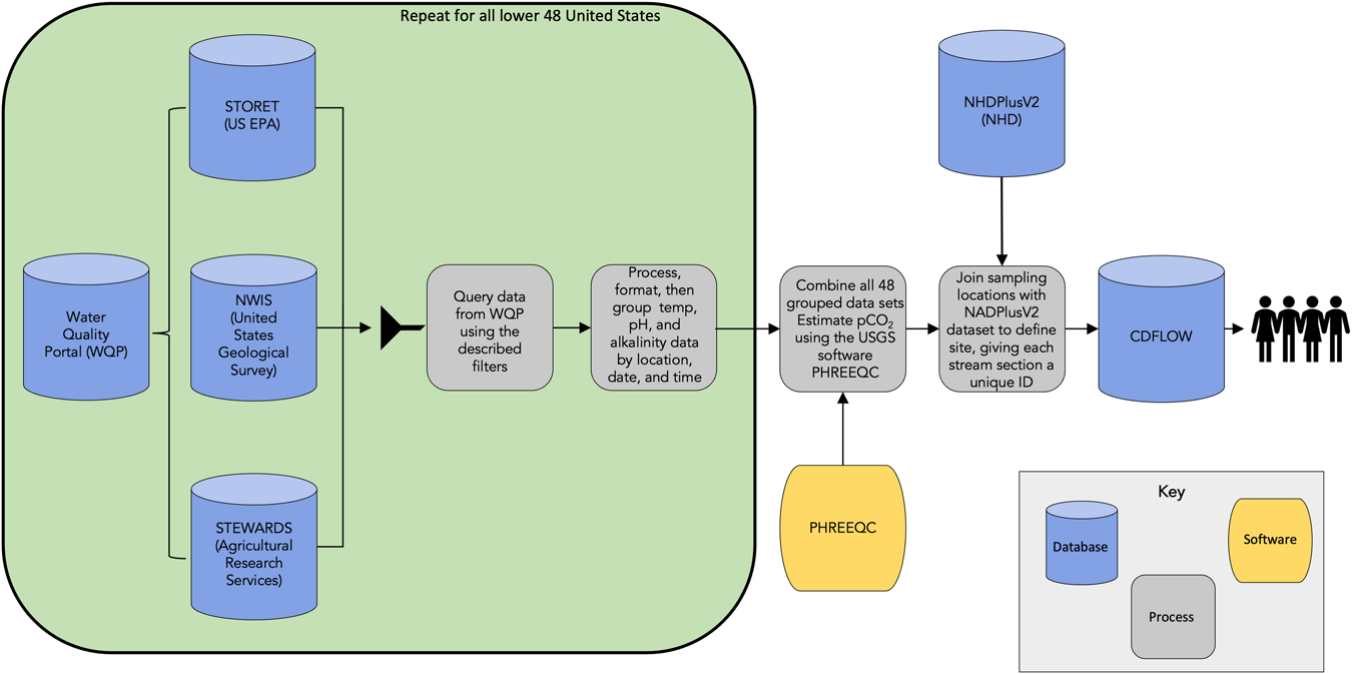
Workflow for developing CDFLOW^19^.

All data queries, manipulations, and calculations were done using the statistical program R version 4.1.2^29^. A visual representation of the workflow to create CDFLOW can be found in Fig. 1.

## Data Records

CDFLOW^19^ exists as a single CSV file that has 779,186 *p*CO_2_ estimates (rows) and 10 variables (columns) across the CONUS from 1990 through 2020 (Table 1). All 48 states within the CONUS are represented across 35,855 sites. CDFLOW^19^ and all supporting code needed to generate and validate the dataset can be downloaded from a public repository on Figshare (10.6084/m9.figshare.19787326).

**Table 1:** Description of the data included in CDFLOW^19^.

Column Names	Date type	Description	Source
Comid	Character	Stream Catchment code where the *p*CO2 estimates exists, code is derived from the EPA StreamCat dataset within the NHD	NHD
Date	Character	Date of temperate, pH and Alkalinity observations	WQP
Time	Character	Time of temperate, pH and Alkalinity observations	
HUC_12	Character	12-digit hydrological unit code	NHD
State	Character	US state where data point was located, given in standard state abbreviation code	WQP
Temp.C	Numeric	Temperature in units Celsius	WQP
pH.std_units	Numeric	pH given in standard pH units	WQP
Alkalinity.ueq/kgw	Numeric	total alkalinity in units - micro equivalence per kilogram water	WQP
*p*CO2.uatm	Numeric	*p*CO2 given in units - micro atmospheres	PHREEQC
CO2.mg/l	Numeric	Concentration of CO_2_ given in milligrams per liter	PHREEQC

Variables are indicated under column names with the data type, a description, and the original source of that column.

**Fig. 2:**
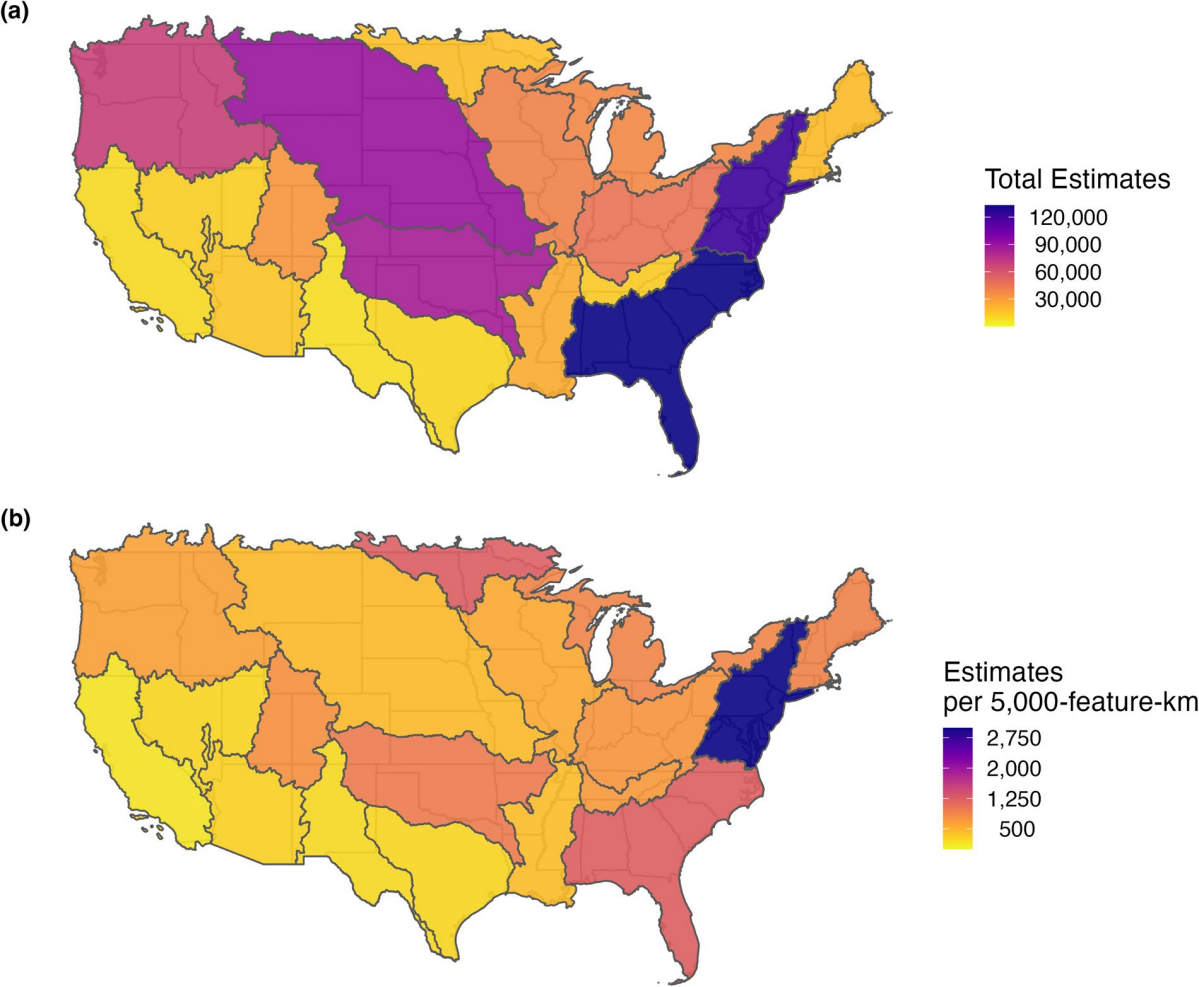
Spatial distribution of *p*CO_2_ estimates within CDFLOW^19^. Panel (**a**) shows the total number of estimates in each Hydrological unit code-2 (HUC2)^28^ within the CONUS. Panel (**b**) shows the total number of estimates divided by the number of 5000-feature-km in each HUC2 within the CONUS.

**Fig. 3:**
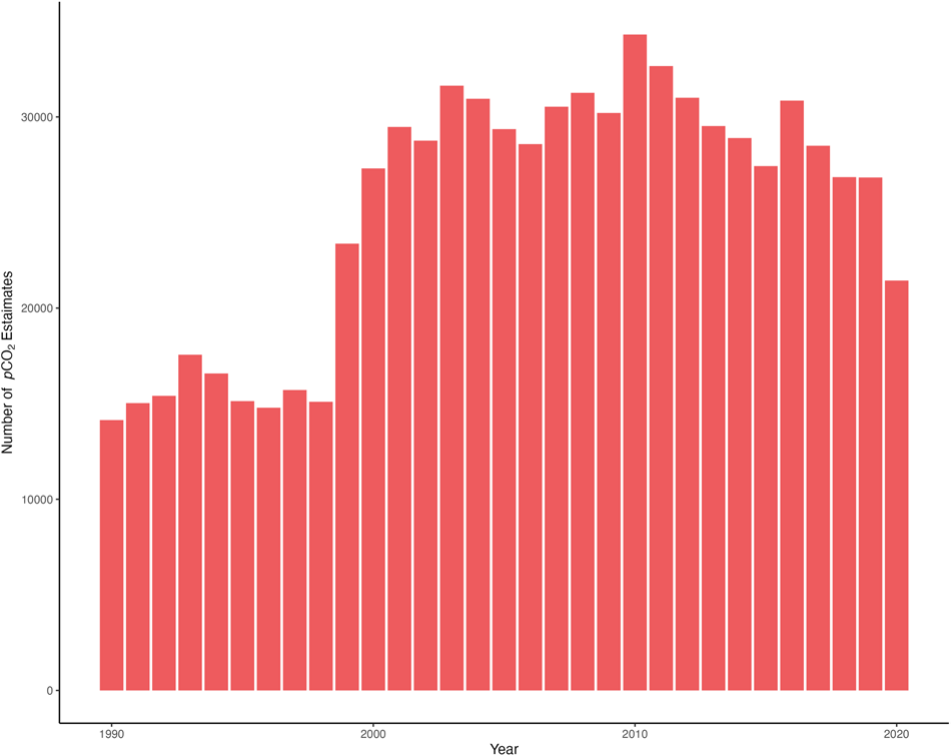
Counts of *p*CO_2_ estimates by year within CDFLOW^19^.

While CDFLOW^19^ has representation across all 48 states and 18 major watersheds within the CONUS, some areas are more represented than others. To display the spatial variability of CDFLOW we grouped estimates by hydrological unit codes (HUC2) and mapped them (Fig. 2). The South Atlantic Gulf and Mid Atlantic Watersheds had the most representation in CDFLOW^19^ followed by the Missouri and Arkansas-White-Red watersheds. Also, we normalized the quantity of estimates within HUC2s by calculating the number of estimates per 5,000 km of stream distance within the HUC2. Total stream distance was calculated by taking the sum of COMID distances within the NHD for each HUC2. The normalized quantity of stream estimates followed similar patterns to the total number of estimates (Fig. 2). Leading us to conclude that estimates are not proportional to quantity of water but other non-environmental factors. We also looked at the temporal scale of CDFLOW^19^ (Fig. 3). Generally, estimates increased going from the 1990’s to the early 2000 were they remained constant then started to decrease from 2015 to 2020. Finally, we inspected spatiotemporal trends of estimates across the CONUS by splitting CDFLOW^19^ into three decades (1990–2000, 2001–2010, 2011–2020). We found that the same spatial trends as the total number of estimates in Fig. 2 held constant across the three decades.

## Technical Validation

### Data validation

*p*CO_2_ values in flowing freshwaters from the literature range widely with typical values falling between 1,300 to 4,300 micro atmospheres, but values in excess of 10,000 micro atmospheres have been reported^30–33^ (micro atmospheres being the unit of the partial pressure of CO_2_). CDFLOW estimates fall within the listed range with mean HUC2 values ranging from 1,200 to 4,500 micro atmospheres and a total interquartile range (25% to 75%) of 1,000 to 3,450 micro atmospheres. Also, CDFLOW does have values that reach in excess of 10,000 micro atmospheres as reported above. Although we find that CDFLOW estimates compare adequately to what is found in the literature, the majority of *p*CO_2_ reported (including those cited here) come from estimated values using similar methods as CDFLOW. In a recent study, Liu *et al*.^34^ assembled a data set of direct measurements of *p*CO_2_ from other published studies. Liu *et al*.^34^ calculated average *p*CO_2_ values in different global ecoregions at 1810, 1540, and 2560 micro atmospheres in the arctic, temperate, and tropics respectively, and again CDFLOW had similar averages.

We downloaded the dataset assembled by Liu *et al*.^34^ and compared it with CDFLOW. However, first, we did the same site join as done in CDFLOW to assign the direct measurements COMIDs and Hydrologic Unit Codes. We then filtered CDFLOW to the months that data from the direct measurements were from and the HUC8s data was located. Both datasets were then filtered so that each HUC8 had a minimum of 10 data points (in order to avoid comparing very low sample sizes). We then did a separate ANOVA comparing the data from CDFLOW and Liu *et al*.^34^ for each HUC8. This resulted in 26 within-HUC8 comparisons. Of those comparisons, less than half (46%) were significantly different (*p* < 0.05), suggesting that most of the time our estimates were distributed the same as those in Liu *et al*. (2022). We also inspected the direction of the bias between the estimates and direct measurements by finding the difference between the median *p*CO_2_ values in each HUC8. This result is akin to examining residuals from a linear model, in which we expect the differences to be centered on 0 and normally distributed. We found that the bias difference (i.e., residuals) between the medians was homoscedastic, which is strong evidence that neither our data or the Liu *et al*.^34^ data was over- or under-estimating *p*CO2.

### Site ground truth

To test the accuracy of the site join procedure used to define sites in CDFLOW we created a procedure to ground truth the site join. The procedure worked by randomly choosing 50 CDFLOW sites and mapping the original latitude and longitude as well as the given COMID *and* all COMID stream features within 0.025 degrees latitude and 0.025 degrees longitude of the original coordinates in 50 separate plots. The resulting 50 plots were then checked manually by 2 observers to demonstrate how often the unsupervised procedure led to a reliable result. Both observers independently found that 50/50 (100%) of the random sites were correctly assigned. The R-script for the analysis is available at the Figshare link (10.6084/m9.figshare.19787326).

### Water quality data portal

The Water Quality Data Portal is a water quality data repository hosted by the United States Geological Survey^22^. Users can interface and download data *via* the Water Quality Data Portal website (https://www.waterqualitydata.us). The Water Quality Data Portal is a dynamic data repository with over 290 million standardized records. A record being a single collected water quality metric. Contributing agencies include all water quality records reported to the United States Geological Survey, the United States Department of Agriculture, and the Environmental Protection Agency.

### National hydrological dataset

The National Hydrological Dataset (NHD) is a national geospatial surface water framework hosted by the Environmental Protection Agency building in conjunction with the United States Geological Survey^20,26^. NHD includes shapefiles mapping all flowing water systems throughout the United States.

### StreamCat

The StreamCat dataset is incorporated into the NHD, which maps stream segments and their associated catchment within the CONUS^27^.

### PHREEQC

PHREEQC Version 3 is a computer program written in the C++ programming language that is designed to perform a wide variety of aqueous geochemical calculations^24^. PHREEQC quantitatively accounts for the chemical composition of a solution by relying on mole-balancing equations. It is free and available (e.g. https://www.usgs.gov/software/phreeqc-version-3).

## Usage Notes

### Estimation uncertainty

PHREEQC relies on the equilibrium of the carbonate system in water in order to estimate *p*CO_2_^25^ and uncertainty has been documented for *p*CO_2_ estimates that rely on carbonate equilibrium. When error is present in *p*CO_2_ estimation using carbonate equilibria, overestimation is usually the error^23,35,36^. We applied filters to data that went into *p*CO_2_ estimation to mitigate overestimation (see methods). Further filters can be applied to data to further mitigate overestimation risks at the discretion of the user; e.g., removing *p*CO_2_ estimates greater than 100,000 parts per million volume, and removing alkalinity values below 1,000 micro equivalents per kilogram water^36^. While absolute values of CDFLOW^19^ *p*CO_2_ estimates may be subject to overestimation relative values and trends are still valid.

Uncertainty estimates from PHREEQC are available as mole balance percent errors. However, when only including three metrics to compute *p*CO_2_ this error term is always quite high but does not necessarily reflect a poor estimate. As discussed in Potter *et al*.^37^ which compares modeled *p*CO_2_ estimates using PHREEQC to direct measurements, they conclude that although mole change balance percent errors are high PHREEQC still provides a good estimate of *p*CO_2_ using pH, temperature, and alkalinity. So, we have decided to exclude mole change balance percent error from the dataset as they are not relevant for modeling purposes and do not negate the validity of CDFLOW *p*CO_2_ estimates.

### Extra parameters

PHREEQC does allow for the inclusion of extra parameters when estimating *p*CO_2_, and more specifically the inclusion of other dissolved inorganic species. However, data on other dissolved inorganic species that matches the same date, time, and location of the pH, temperature, and alkalinity is only available to a limited number of observations. Due to the limited number of other dissolved inorganic species for observation they were excluded from the PHREEQC estimation. However, the use of other dissolved inorganic species in estimating *p*CO_2_ using PHREEQC would potentially allow for more robust estimates. If CDFLOW users are interested in the inclusion of other dissolved inorganic species a supporting script can be found at the Figshare link (10.6084/m9.figshare.19787326) that describes and gives examples of the changes required to do so.

### Expanding data

By defining sites in CDFLOW^19^ by which COMID they fall into gives each site all the data that corresponds to that COMID. COMID data can be accessed *via* the NHD (see technical validation). COMID data can also be accessed *via* R package NHD Tools^38^.

## Data Availability

Code for the creation of CDFLOW is available as a series of R scripts *via* public repository on Figshare^19^ (10.6084/m9.figshare.19787326).
